# Structural basis for electron transport mechanism of complex I-like photosynthetic NAD(P)H dehydrogenase

**DOI:** 10.1038/s41467-020-14456-0

**Published:** 2020-01-30

**Authors:** Xiaowei Pan, Duanfang Cao, Fen Xie, Fang Xu, Xiaodong Su, Hualing Mi, Xinzheng Zhang, Mei Li

**Affiliations:** 1 National Laboratory of Biomacromolecules, CAS Center for Excellence in Biomacromolecules, Institute of Biophysics, Chinese Academy of Sciences, Beijing, 100101 PR China; 2 University of Chinese Academy of Sciences, Beijing, 100049 PR China; 3 National Key Laboratory of Plant Molecular Genetics, Institute of Plant Physiology and Ecology, Shanghai Institutes for Biological Sciences, Chinese Academy of Science, Shanghai, 200032 PR China; 4 Center for Biological Imaging, CAS Center for Excellence in Biomacromolecules, Institute of Biophysics, Chinese Academy of Sciences, Beijing, 100101 PR China

**Keywords:** Membrane proteins, Cryoelectron microscopy

## Abstract

NAD(P)H dehydrogenase-like (NDH) complex NDH-1L of cyanobacteria plays a crucial role in cyclic electron flow (CEF) around photosystem I and respiration processes. NDH-1L couples the electron transport from ferredoxin (Fd) to plastoquinone (PQ) and proton pumping from cytoplasm to the lumen that drives the ATP production. NDH-1L-dependent CEF increases the ATP/NADPH ratio, and is therefore pivotal for oxygenic phototrophs to function under stress. Here we report two structures of NDH-1L from *Thermosynechococcus elongatus* BP-1, in complex with one Fd and an endogenous PQ, respectively. Our structures represent the complete model of cyanobacterial NDH-1L, revealing the binding manner of NDH-1L with Fd and PQ, as well as the structural elements crucial for proper functioning of the NDH-1L complex. Together, our data provides deep insights into the electron transport from Fd to PQ, and its coupling with proton translocation in NDH-1L.

## Abstract (web summary)

NAD(P)H dehydrogenase-like complex NDH-1L couples the electron transport from ferredoxin (Fd) to plastoquinone (PQ) and proton pumping from cytoplasm to the lumen. Here authors report two structures of NDH-1L from *Thermosynechococcus elongatus* BP-1, in complex with one Fd and an endogenous PQ, respectively.

## Introduction

During oxygenic photosynthesis, light energy is utilized by photosystems I and II (PSI and PSII) to drive the electron transport, which can be classified into two types, the linear electron transport/flow and the cyclic electron transport/flow^1,2^. During LEF, electrons are extracted from water molecules by PSII and transported to PSI through cytochrome *b*_*6*_*f* (Cyt*b*_*6*_*f*) and free electron carrier including plastoquinone (PQ), before being ultimately used to reduce NADP^+^ to NADPH. Simultaneously, a proton gradient across the thylakoid membrane is generated and drives the ATP synthase to produce ATP^3^. Both NADPH and ATP are required for the subsequent CO_2_ assimilation during the Calvin–Bensen–Bassham cycle and other cellular metabolisms^2,3^. During the CEF process, the electrons are recycled between PSI and Cyt*b*_*6*_*f* through PQ pool. CEF functions without PSII, simply creating the transmembrane proton gradient, which allows only ATP production without generating NADPH^4,5^. LEF alone probably generates insufficient ATP to balance the ATP/NADPH consumption for CO_2_ assimilation^6–8^, whereas CEF increases the produced ATP/NADPH ratio^9^, thus is important for achieving high efficiency of the Calvin–Bensen–Bassham cycle, especially under multiple stressed conditions^4,10–12^, as well as for C4 photosynthesis^13,14^. In addition, photosynthetic organisms that are exposed to environmental stress usually exhibit increased demand for ATP^15^, therefore, CEF is crucial for the quick response of phototrophs to the changing environment. Furthermore, CEF plays an essential role in photoprotection by generating a large transmembrane proton gradient, thereby inducing energy dependent quenching to dissipate excessively absorbed energy^9,16–19^.

In both cyanobacteria and plants, a type-I NAD(P)H dehydrogenase-like (NDH-1) complex is crucial for CEF and respiration. NDH-1 complex accepts electrons from electron carrier ferredoxin (Fd) or NAD(P)H in PSI acceptor side, and transfers them to Cyt*b*_*6*_*f* through PQ^20,21^. NDH-dependent CEF was previously suggested to fine-tune the chloroplast redox state, and to prevent overreduction of the stroma under stress^2,14^. The NDH-1 complexes belong to the NAD(P)H:Quinone oxidoreductase family that includes the complex I (NADH dehydrogenase) of the respiratory chain in bacteria and mitochondria^15,22^. Cyanobacteria possess several types of NDH-1 complexes, namely NDH-1L/1L′ (large), NDH-1M (medium), and NDH-1S/1S′ (small), differing in size and the isoforms of NdhD/NdhF subunits, and the latter two types can further associate into NDH-1MS/NDH-1MS′^23,24^. NDH-1L containing NdhD1/NdhF1 is involved in CEF around PSI and respiration^15,20^. The NDH-1L complex and bacterial complex I form a similar L-shaped architecture and share 11 conserved subunits. In addition, cyanobacterial NDH-1L possesses eight oxygenic photosynthesis-specific (OPS) subunits^25^, namely NdhL^26^, NdhM, NdhN^27^, NdhO^28^, NdhP, NdhQ^29^, NdhS^30^, and NdhV^31,32^, but lacks three hydrophilic subunits, which contain the NADH binding site in bacterial complex I^33^. These earlier results were confirmed by recent studies showing that the photosynthetic NDH complex binds Fd as the electron donor, rather than NADPH^34,35^. The electron delivered from Fd is eventually transferred to PQ which is bound within the NDH complex.

Two structures of NDH-1L from the thermophilic cyanobacterium *Thermosynechococcus elongatus* BP-1 were recently reported^35,36^. These studies identified the structures and locations of 18 subunits (lacking NdhV), providing solid foundations for better understanding the architecture and assembly of the NDH-1L complex. NDH-1L in these two structures adopts a characteristic L-shaped architecture, which can be divided into membrane arm/domain and hydrophilic arm/domain, composed of ten and eight subunits, respectively. However, the structure and accurate location of the soluble subunit NdhV, which might be loosely associated with the complex^31,32^, remain unknown. Moreover, it was previously suggested that NdhS participates in Fd binding^34,37^, while another recent study proposed an alternative Fd binding pocket formed by NdhO^36^. To date, the exact Fd binding site in cyanobacterial NDH-1L remains to be elucidated. In addition, as only the apo form of NDH-1L was structurally characterized, the detailed interactions of PQ within photosynthetic NDH complex has not been determined, although a quinone-reaction chamber (Q chamber) was previously suggested in respiratory complex I, based on the substrate/inhibitor-bound structures from *Thermus thermophilus* (Tt complex I)^38^ and *Yarrowia lipolytica* (Yl complex I)^39^. To further advance our understanding of NDH functionality, we solved two single particle cryo-electron microscopy (cryo-EM) structures of NDH-1L from *T. elongatus* BP-1. Our results provide the structural model of NDH-1L containing NdhV, and reveal the detailed binding modes of Fd and PQ. Moreover, we observe a variety of conformational changes between our structure and previously reported NDH/complex I structures, thus shedding light on the electron transport process and its coupling with proton translocation.

## Results

### Overall structure

**Fig. 1:**
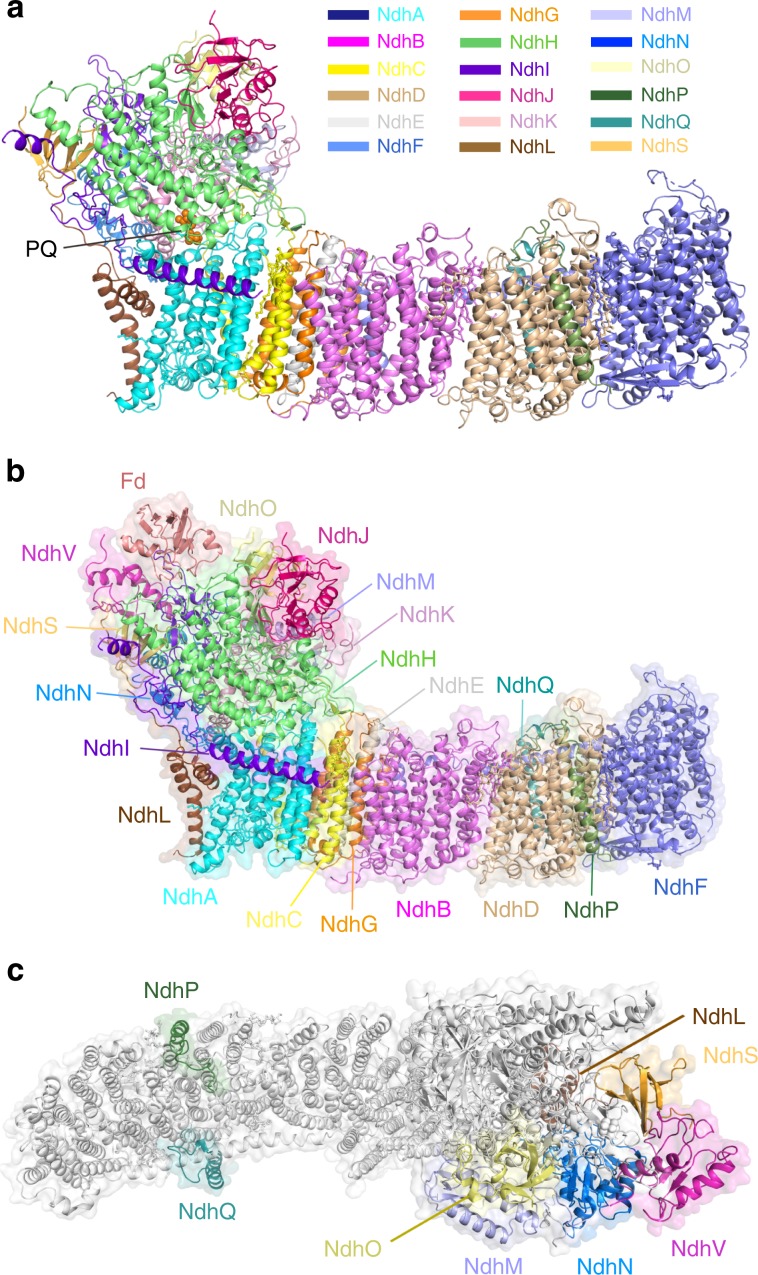
The NDH-PQ and NDH-Fd structures. **a** Cartoon representation of the NDH-PQ structure viewed along the membrane plane. PQ is shown as orange spheres and indicated. **b** The overall structure of NDH-Fd viewed along the membrane plane. **c** The location of OPS subunits in NDH-1L complex. The eight OPS subunits are colored differently and indicated, while other subunits are shown in white.

We first solved the structure of NDH-1L complex purified from *T. elongatus* BP-1 (Supplementary Fig. 1) at an overall resolution of 3 Å (Supplementary Fig. 2, Table 1). Interestingly, we found that the NDH-1L complex binds a PQ molecule at a pocket above the membrane plane (Fig. 1a). We assume that this is an endogenous PQ molecule, which was trapped in the cyanobacterial NDH-1L complex during purification, as was earlier reported for *Escherichia coli* complex I^40^. Thus, we named this structure as NDH-PQ. Similar to the two previously reported cyanobacterial NDH-1L structures^35,36^, our NDH-PQ structure also contains 18 subunits (Fig. 1a), without any signs of association with NdhV. These results indicated that NdhV is loosely associated with other NDH subunits, in line with previous biochemical data^31,32^. In addition, we found that the NdhS subunit in the NDH-PQ structure exhibits weak cryo-EM density, suggesting that NdhS is also loosely associated. Previous reports showed that NdhV interacts with NdhS and requires NdhS for its stabilization^31,32^. Therefore, to obtain the intact NDH-1L in complex with Fd, we expressed and purified the proteins of Fd, NdhV, and NdhS from *T. elongatus* BP-1 via *E. coli* (Supplementary Fig. 1) and incubated the purified proteins with NDH-1L in order to increase their occupancy, and finally solved the structure of NDH-1L containing NdhV and in complex with Fd (NDH-Fd) at 3.2 Å resolution (Supplementary Fig. 3, Table 1). The NDH-Fd structure, highly similar with the NDH-PQ structure, contains all 19 NDH subunits, and binds one Fd molecule at the top of the hydrophilic arm (Fig. 1b). Supplementary Fig. 4 shows the representative cryo-EM densities of Fd and several subunits and cofactors of NDH-1L complex.

**Table 1:** Cryo-EM data collection and refinement and validation statistics.

	NDH-PQ (EMDB-9990, PDB 6KHJ)	NDH-Fd (EMDB-9989, PDB 6KHI)
Data collection and processing		
Magnification	130,000	130,000
Voltage (kV)	300	300
Electron exposure (e^−^/Å^2^)	60	60
Defocus range (μm)	0.5–2.5	0.5–2.5
Pixel size (Å)	1.04	1.04
Symmetry imposed	C1	C1
Initial particle images (no.)	434,021	276,859
Final particle images (no.)	187,788	152,003
Map resolution (Å)	3.0	3.2
FSC threshold	0.143	0.143
Map resolution range (Å)	2.5–4.5	2.8–4.4
Refinement		
Initial model used (PDB code)	4HEA	NDH-PQ, 5AUI
Model resolution (Å)	3.0	3.2
FSC threshold	0.5	0.5
Map sharpening *B* factor (Å^2^)	−98	−106
Model composition		
Nonhydrogen atoms	31,027	32,635
Protein residues	3877	4095
Ligands	20	19
B factors (Å^2^)		
Protein	45.0	55.6
Ligand	48.5	60.4
R.m.s. deviations		
Bond lengths (Å)	0.007	0.006
Bond angles (°)	0.999	0.945
Validation		
MolProbity score	1.86	1.92
Clash score	6.84	7.31
Poor rotamers (%)	0.41	0.27
Ramachandran plot		
Favored (%)	92.13	91.04
Allowed (%)	7.85	8.94
Disallowed (%)	0.02	0.02

### The membrane arm with lipid and carotenoid molecules

**Fig. 2:**
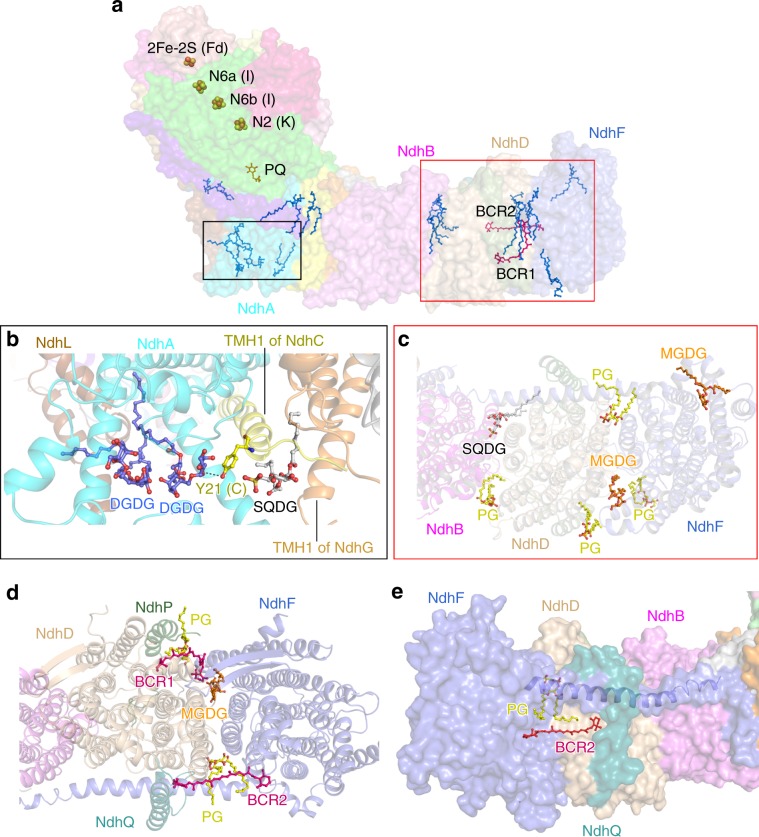
The membrane arm with lipid and carotenoid molecules. **a** Distribution of cofactors in NDH-Fd structure. Fe–S clusters are shown as spheres, PQ (orange), lipids (blue), and carotenoids (red) are shown as sticks. **b** Three lipid molecules (shown as ball-and-stick mode) contribute to the stabilization of NdhL, NdhA, NdhC, and NdhG. **c** The stromal side view of the lipid distribution at the distal part of the membrane arm. **d** Two carotenoid (BCR) and three lipid molecules participate in NdhF–NdhD interactions. **e** The binding pocket of BCR2. The color codes of subunits are the same as those in Fig. 1. The horizontal helix of NdhF is shown as blue cartoon. The BCR molecules are shown as red sticks. Lipid molecules are colored yellow for phosphatidyl glycerol (PG), gray for sulfoquinovosyldiacyl glycerol (SQDG), orange for monogalactosyldiacyl glycerol (MGDG), and blue for DGDG in **b**–**e**.

The membrane arm in NDH-PQ and NDH-Fd structures are almost identical, seven subunits (NdhA-G) conserved in respiratory complex I constitute the membrane core, and three OPS subunits (NdhL, NdhP-Q) attach to the peripheral region. NdhP and NdhQ are located at the distal part, sandwiching NdhD from opposite sides, whereas NdhL is positioned adjacent to NdhA (Fig. 1c). Furthermore, we modeled two carotenoids and thirteen lipid molecules (Supplementary Fig. 4g–l) within the membrane arm in both NDH-PQ and NDH-Fd structures (Fig. 2). In addition to the previously described protein–protein interaction^35,36^, these nonprotein components greatly contribute to the integrity of the membrane arm. At the proximal region, two digalactosyldiacyl glycerol (DGDG) molecules fill the empty space between NdhA and NdhL from the luminal side, with one DGDG further hydrogen bonded with the N-terminal region of NdhC. In the vicinity of the two DGDGs, another lipid molecule is sandwiched by the first transmembrane helix (TMH) of NdhC and NdhG. Therefore, the three lipid molecules function in linking the three proximal subunits at the lumen (Fig. 2b). At the distal region, seven lipid molecules were observed, and six are located at the stromal side (Fig. 2a). Among the seven lipids, four and two are found to gather at the NdhF–NdhD, NdhD–NdhB interfaces, respectively (Fig. 2c). Moreover, two carotenoid (modeled as β-carotene) molecules greatly enhance the NdhF–NdhD interaction. One β-carotene (BCR1) is located near NdhP and sandwiched by two lipid molecules (Fig. 2d), together forming a cluster and serving as molecular glue, as was previously suggested by a NDH-1L structural study^35^. On the opposite side close to NdhQ, the second β-carotene (BCR2) molecule is almost parallel to the membrane plane and binds in a pocket below the horizontal helix of NdhF (Fig. 2e). This observed β-carotene secures the NdhF–NdhD interaction, comparable to a bolt-like feature. In addition to their function for light harvesting and energy quenching, carotenoid molecules are also crucial for the inter-subunit interaction and stabilization for the photosynthetic complexes^41^. The two β-carotene molecules result in the strong association between NdhF and NdhD, thus explaining the presence of a smaller NDH-1M complex in cyanobacteria, which lacks both NdhD and NdhF subunits^42,43^. In addition, our structures indicate that both NdhP and NdhQ form direct contacts with NdhD and NdhF, and further interact with interfacial lipids and carotenoids (Fig. 2d), thus contribute to the stabilization of NDH-1L complex, in line with the finding from previous reports that deletion of each subunit disassembles the NDH-1L complex^44,45^.

### The hydrophilic arm and the interface between two arms

**Fig. 3:**
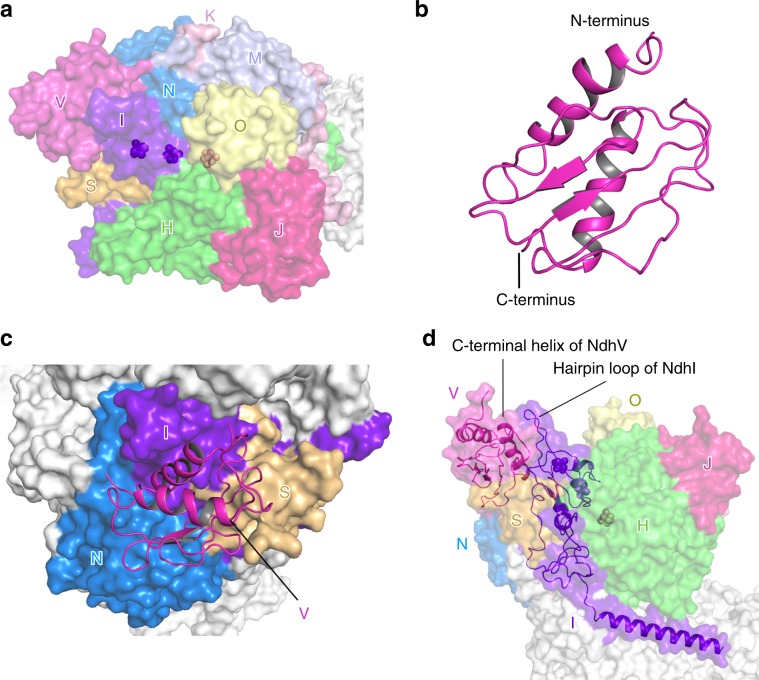
The hydrophilic arm containing NdhV. **a** The location of subunits in the hydrophilic domain. The nine NDH subunits in the hydrophilic domain are colored differently and labeled with the abbreviations of one letter (for example, letter V indicates subunit NdhV). The 4Fe–4S clusters are shown as spheres. **b** Overall structure of NdhV. **c** The binding position of NdhV. NdhV is shown in cartoon, other subunits are shown in surface mode. NdhI, NdhS, and NdhN are colored differently and labeled, whereas other subunits are shown in white. **d** The C-terminal helix of NdhV interacts with the hairpin loop of NdhI.

The NDH-Fd structure contains the complete hydrophilic arm composed of nine subunits, including NdhV (Fig. 3a), which was absent in both previously reported NDH structures. The four subunits conserved in respiratory complex I (NdhH-K) form the hydrophilic core of NDH-1L. Among them, NdhI and NdhK harbor two and one 4Fe–4S clusters (corresponding to the clusters N6a, N6b, and N2 from the respiratory complex I) essential for electron transport (Figs. 2a, 3a). Five OPS subunits (NdhM, NdhN, NdhO, NdhS, and NdhV) are located at the peripheral region, surrounding half of the hydrophilic core (Fig. 1c). Extensive interactions are formed between the conserved subunits and OPS subunits.

NdhV is composed of central loops/β-strands, which is flanked by the N- and C-terminal helix at one side (Fig. 3b, Supplementary Fig. 4a). In the NDH-Fd structure, NdhV is located above NdhN and NdhS (Fig. 3c). The central loops/β-strands of NdhV join with the loops/β-strands of NdhS (Supplementary Fig. 5a), forming strong interactions. This structural observation is in line with previous results suggesting that NdhV requires NdhS for its binding and stabilization^31,32^. In addition, NdhV, NdhS, and NdhN contact each other and the three OPS subunits together form a triangular structure, simultaneously interacting with NdhI (Supplementary Fig. 5b). Particularly, NdhV uses its C-terminal helix to associate with the long hairpin loop of NdhI (Supplementary Fig. 5c), which projects into the cytoplasmic region above the hydrophilic arm (Fig. 3d). The close association between NdhV and NdhI observed in the structure agrees with an earlier report that NdhV is crosslinked with NdhI^31^.

In NDH complex, the soluble subunits NdhK and NdhH together with the membrane subunit NdhA play major role in maintaining the association between the membrane and hydrophilic arms (Supplementary Fig. 6a). In addition, NdhI, NdhC, and the OPS subunit NdhL also contribute to the interaction between the two arms. The cytoplasmic loop between the first and second TMH (TMH1-2 loop) of NdhC (Supplementary Fig. 4d) contacts NdhA and the soluble subunits NdhK/NdhH/NdhM (Supplementary Fig. 6b), reinforcing the interactions between the two arms. Moreover, the membrane subunit NdhL has a C-terminal fragment projecting into the cytoplasmic region and associating with NdhI/NdhN (Supplementary Fig. 6c). While the soluble NdhI possesses an N-terminal amphiphilic helix (N-helix) that embraces the membrane subunits NdhA/NdhL (Supplementary Fig. 6c). In addition, three lipid molecules were found at the interface between the two arms, facilitating their association (Supplementary Fig. 6d, e). Two of these lipids link the N-helix of NdhI with the membrane domain of NdhA and NdhC (Supplementary Fig. 6d), whereas the third lipid is located in an enclosed cavity formed by the N-terminal fragments from NdhL, NdhN, and NdhK (Supplementary Fig. 6e). Our structure highlights the pivotal role of lipid molecules in connecting the hydrophilic arm with the membrane arm of the NDH complex.

### The Fd binding pocket

**Fig. 4:**
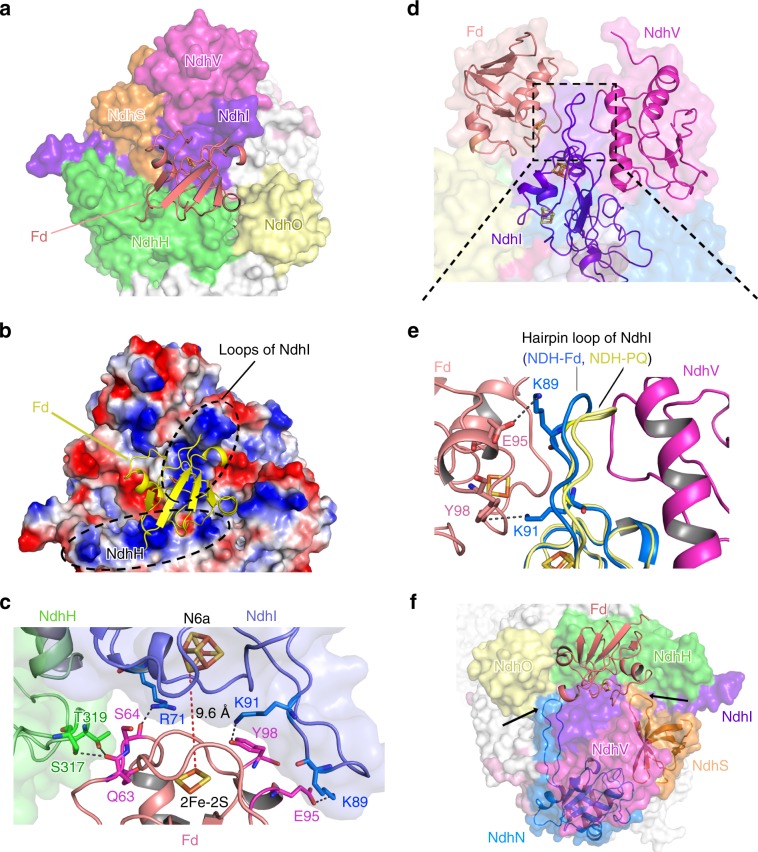
The Fd binding pocket. **a** The Fd binding pocket is mainly formed by NdhI and NdhH. Fd is shown in cartoon, the NDH subunits are shown in surface mode. Subunits close to Fd are colored differently and other subunits are shown in white. **b** The electrostatic surface representation of Fd binding pocket. Red represents negative charge, and blue represents positive charge. Fd is shown as yellow cartoon. The positively-charged regions surrounding Fd are highlighted with black dashed circle and labeled. **c** The interactions between Fd and NdhI/NdhH subunits. The Fe–S clusters and the residues involved in the interactions between Fd and NdhI/NdhH are depicted as sticks and labeled. The hydrogen bonds are shown as black dashed lines. The distance between Fe–S clusters of NdhI and Fd (red dashed line) is indicated. **d** Fd and NdhV almost symmetrically bind the hairpin loop of NdhI from the opposite sides. The zoom-in view of the region circled by the black dashed line is shown in (**e**). **e** Superposition of NDH-Fd and NDH-PQ structures showing the different conformation of the hairpin loop in NdhI subunit. Two positively-charged Lys residues from the hairpin loop of NdhI are hydrogen bonded with residues from Fd. Residues involved in the interactions are shown as sticks and labeled. **f** The loop regions in NdhN and NdhS (pointed with black arrows) interact with NdhI and/or NdhH and help to stabilize the Fd binding pocket.

Several OPS subunits, including NdhS, NdhO, and NdhV, were previously suggested to be involved in Fd binding^32,34,36^. Surprisingly, neither of these subunits directly interact with Fd as shown in our NDH-Fd structure, whereas two conserved subunits, NdhI and NdhH, form extensive contacts with Fd. The long hairpin loop of NdhI, together with a C-terminal fragment from NdhH, shapes a positively-charged patch on the surface of NDH-1L complex, and forms strong electronic interactions with the acidic residues of Fd (Fig. 4a–c), thus is essential for Fd binding. This structural feature is consistent with previous studies showing that Fd features a highly acidic surface, with recruitment usually driven by basic patches on the binding partner^46–48^. Furthermore, our surface plasmon resonance (SPR) results demonstrated that the binding affinity of NDH-1L with Fd is significantly increased at higher pH (Supplementary Fig. 7), probably because the stronger interaction between the acidic Fd and the basic NDH complex.

In addition to NdhI and NdhH, several OPS subunits also contribute to the Fd binding, though they lack any direct contacts with Fd. In our NDH-Fd structure, NdhV and Fd are almost symmetrically distributed along opposite sides of the hairpin loop of NdhI (Fig. 4d), thus NdhV provides strong support for the hairpin loop. Two positively-charged residues, which are located on the hairpin loop and highly conserved in NdhI, are hydrogen bonded with residues from Fd. Superposition of our NDH-Fd and NDH-PQ structures revealed a swing of the hairpin loop toward Fd in NDH-Fd structure (Fig. 4e). This observation suggests NdhV may enhance the NdhI–Fd interaction by simultaneously binding the long hairpin loop of NdhI, which provides a rationale for previous data suggesting that NdhV is pivotal for CEF activity^31,32^. Moreover, by contacting NdhI and/or NdhH through their loop regions, NdhS and NdhN may also contribute to the formation of the Fd binding pocket (Fig. 4f). The interactions between Fd and NDH complex position the 2Fe–2S cluster of Fd close to the N6a cluster of NdhI, with a distance of 9.6 Å (Fig. 4c). This distance is comparable to those between other 4Fe–4S clusters in NDH-1L complex, and could enable efficient electron transport^49,50^.

### The PQ chamber

**Fig. 5:**
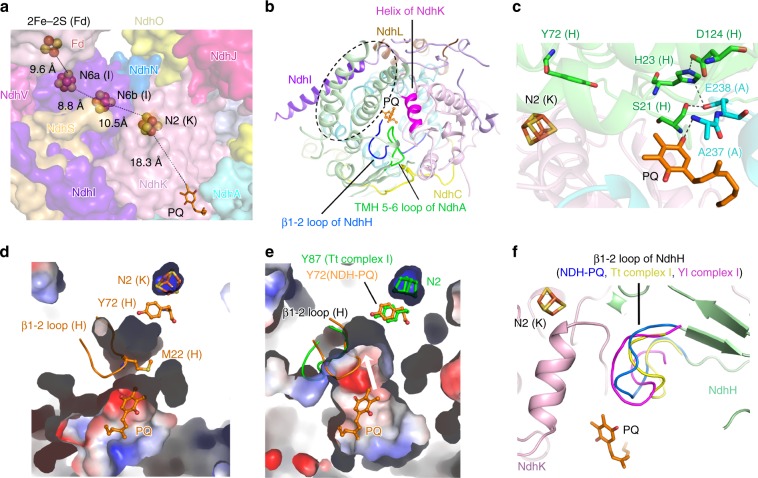
The PQ chamber. **a** The cofactors involved in the electron transport chain. The PQ molecule is shown as sticks and the Fe–S clusters are shown as spheres, and the distances between cofactors are indicated. The subunits around PQ chamber are shown in surface with the same color code as that in Fig. 1. NdhH subunit is omitted for clarity. **b** The PQ chamber is shaped by NdhH, NdhK, and NdhA, which are further stabilized by NdhI, NdhL, and NdhC. The structural elements involved in forming the PQ chamber are indicated. The four-helix bundle of NdhH is highlighted with black dashed circle. The PQ molecule is shown in ball-and-stick mode and colored orange. **c** The interactions around PQ molecules. The N2 cluster, PQ, and key residues are shown in sticks and labeled. Comparison of the PQ chamber in NDH-PQ structure (**d**) and the Q chamber in Tt complex I structure (PDB ID: 4HEA) (**e**) in the same view. In NDH-PQ structure, the β1-2 loop containing M22 of NdhH intrudes into the PQ chamber and blocks the path from PQ to Y72 of NdhH and N2 cluster of NdhK, resulting in a smaller occluded PQ chamber (**d**), while the corresponding loop in Tt complex I structure (colored green) adopts a different conformation and results in a larger opened Q chamber (**e**). The NDH-PQ structure (colored orange) is superposed to Tt complex I structure (colored green) in **e**, to show the positions of β1-2 loop and Y72 of NdhH as well as the PQ molecule in NDH-PQ structure. The white arrow indicates that a quinone molecule at the PQ position is able to move toward the N2 cluster in the larger PQ pocket as shown in the Tt complex I structure. **f** Comparison of the PQ/Q chambers in NDH-PQ, Tt complex I (PDB ID: 4HEA), and Yl complex I (PDB ID: 4WZ7) structures. The β1-2 loop of NdhH in NDH-PQ structure and the corresponding loops in Tt and Yl complex I structures are highlighted in different colors and labeled.

In the NDH-PQ structure, we modeled a PQ molecule in a pocket (PQ chamber) at the interface of the membrane and hydrophilic arms (Fig. 1a). It is noteworthy that the PQ molecule exhibits weaker density compared with other stable parts of the NDH structure, indicating that either the PQ molecule is mobile or a portion of NDH complex in our preparation does not bind PQ. The head group of PQ is oriented toward the N2 cluster (Fig. 5), and the end of its tail is invisible in the cryo-EM map (Supplementary Fig. 4f). The four-helix bundle region and the loop between the first two β-stands (β1-2 loop) of NdhH, the fragment around the second helix of NdhK, and the loop connecting TMHs 5 and 6 (TMH5–6 loop) of NdhA are the major structural elements that constitute the PQ chamber (Fig. 5b). These fragments are further stabilized by subunits NdhI, NdhL, and NdhC (Fig. 5b). Interestingly, these six subunits are also essential for maintaining the association between the membrane and hydrophilic arms (Supplementary Fig. 6a–c). Within the chamber, the head group of PQ molecule is located ~18 Å from the N2 cluster (Fig. 5a). The PQ interacts with NdhA-A237 through one of its ketone group (Fig. 5c). In addition, E238 of NdhA is hydrogen bonded with residues from β1-2 loop of NdhH, including H23, which is further hydrogen bonded with NdhH-D124 (Fig. 5c). The PQ chamber is located at an area similar to that of the Q chamber identified from Tt complex I structure^38^, but the PQ chamber in our structure is smaller than the previously assigned Q chamber at the upper part close to the N2 cluster (Fig. 5d, e). The smaller PQ chamber is caused by the different conformation adopted by the β1-2 loop of NdhH, which intrudes into the PQ chamber, and the side chain of M22 blocks the path between PQ and the N2 cluster (Fig. 5d, f). In comparison, the corresponding β1-2 loop in Tt complex I structure is away from the Q chamber, resulting in a larger chamber that is accessible to the N2 cluster (Fig. 5e, f). In agreement with that, the substrate/inhibitor in Q chamber in Tt complex I is located only ~12 Å from the N2 cluster, and interacts with residues Y87 and H38 of Nqo4 subunit (corresponding to NdhH-Y72 and NdhH-H23)^38^. These two residues were previously suggested to protonate quinone during the catalytic cycle. In addition, residue D139 of the Nqo4 subunit (corresponding to NdhH-D124) forms a hydrogen bond with H38, facilitating the protonation process^38^. These residues are completely conserved in the cyanobacterial NDH-1L, implying that similar interactions can also be formed in NDH-1L complex. Indeed, the H23-D124 hydrogen bond is preserved in our NDH-PQ structure (Fig. 5c); however, the PQ molecule is more distantly located from the N2 cluster, without direct linking with NdhH-Y72 and NdhH-H23, implying that our structure represents a state of NDH/complex I that is not fully active. In addition to the β1-2 loop of NdhH, the TMH5–6 loop of NdhA in our NDH structure and the corresponding loops in complex I structures also exhibit variable conformations (Supplementary Fig. 8a). Moreover, the TMH5–6 loop of NdhA was not completely built in our NDH structure due to the very weak densities of a short fragment (residues L234–E236). Together, these results suggest that the two loops are highly flexible, and that they may adopt various conformations at different states and/or under different conditions.

### Comparison of three NDH-1L structures

Together with the work presented here, three structures of NDH-1L with identical subunit composition are now available, with two from previous works (PDB codes 6HUM and 6NBY)^35,36^. Interestingly, the three NDH complexes were purified under different pH conditions, thus these three structures are termed based on their purified pH values, namely NDH_pH7_ for our NDH-PQ structure, and NDH_pH8_ (6HUM)^35^ and NDH_pH6_ (6NBY)^36^ for the previously reported structures. Structural comparison showed that the two loops around the PQ chamber, TMH5–6 loop of NdhA and β1-2 loop of NdhH, also adopt different conformations among the three NDH structures (Supplementary Fig. 8b). This result indicates the flexible nature of the loops constituting the PQ chamber (Q chamber), which might be associated with the physiological function of NDH-1L.

**Fig. 6:**
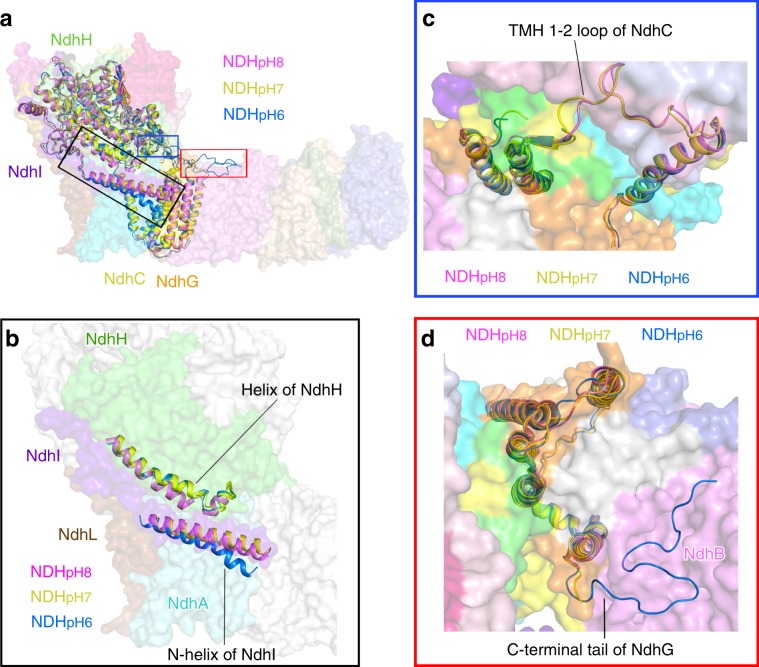
Comparison of three NDH-1L structures under different pH conditions. **a** Comparison of NDH-PQ structure (NDH_pH7_) with previously reported two NDH-1L structures NDH_pH6_ and NDH_pH8_ (PDB codes: 6NBY and 6HUM) superposed on NdhA subunit. The subunits with structural differences are depicted as cartoons and labeled. The regions with evident differences among the three structures are circled with black, blue, and red boxes. **b** Comparison of the NdhI and NdhH subunits. The N-helix of NdhI and a helix of NdhH are highlighted as cartoons. The subunits similar in three NDH-1L structures are shown as surface mode (For clarity, only NDH_pH7_ structure is shown). **c** Comparison of NdhC subunits viewed from the stromal side. The TMH1-2 loop was not modeled in NDH_pH6_ structure. **d** Comparison of NdhG subunits viewed from the stromal side. Its C-terminal tail in NDH_pH6_ structure is almost completely modeled and interacts with the stromal region of NdhB. Color codes are the same as that in Fig. 1.

Structural comparison showed that NDH_pH7_ and NDH_pH8_ structures are relatively similar with each other, whereas NDH_pH6_ structure exhibits distinct local conformation of several subunits, which mainly occur at the interface between the membrane arm and the hydrophilic arm (Fig. 6a). The amphiphilic N-helix of NdhI stabilizes the membrane arm and simultaneously interacts with the soluble subunit NdhH. The distance between NdhH and the N-helix of NdhI is the shortest in the NDH_pH8_ structure, but the longest in the NDH_pH6_ structure (Fig. 6b). Moreover, the TMH1-2 loop of NdhC, which is pivotal for connecting the membrane and the hydrophilic arms (Supplementary Fig. 6b) was well resolved, exhibiting similar conformations in NDH_pH7_ (Supplementary Fig. 4d) and NDH_pH8_ structures, but is invisible in NDH_pH6_ structure (Fig. 6c). In contrast to the TMH1-2 loop of NdhC, the C-terminal fragment of NdhG in NDH_pH6_ structure is considerably stable and almost completely modeled. This fragment forms extensive hydrogen bonds with stromal residues of NdhB. In comparison, the C-terminal 30 residues of NdhG were not built in the other two structures (Fig. 6d). The structural comparison revealed that the NDH-1L is more rigid in the membrane arm at low pH, whereas the complex is more strongly associated between its two arms at higher pH.

To evaluate the influence of pH on the function of NDH-1L, we further tested the CEF activity of the NDH-1L complex under various pH conditions (Supplementary Fig. 9). The CEF activity was monitored by measuring the postillumination increase in chlorophyll fluorescence, a method extensively used in previous reports^31,32,51–54^. Our results showed that NDH-1L exhibits the highest activity in mediating the CEF at pH 8.0 and pH 7.0, while at pH 6.0, its activity drastically decreased (Supplementary Fig. 9c). These results implied the possibility that the close association between the membrane arm and the hydrophilic arm is related to the functionality of NDH-1L complex.

## Discussion

The cryo-EM reconstructions of cyanobacterial NDH-1L complex presented here provide the complete structural model of NDH-1L and reveal the detailed binding pockets of Fd and PQ. Our structural analysis suggested that NdhV regulates the binding of Fd with NDH complex. In the NDH-Fd structure, NdhV bound to one side of the hairpin loop of NdhI may buttress the binding of Fd with NdhI at the opposite side (Fig. 4d, e). Thus, NdhV and Fd may bind NdhI in a synergistic manner. Previous report indicated that the level of NdhV is upregulated at high light^31^, which may increase the percentage of the NDH-1L complex containing NdhV, and further result in more Fd-bound NDH-1L. This can explain previous study showing that NDH-1 exhibits higher CEF activity at high light, which was suppressed by deletion of NdhV^31^. Therefore, the association of NdhV may fine-tune the activity of NDH complex by regulating its binding affinity with Fd.

The PQ chamber observed in our structure is located in an area close to the Q chamber in respiratory complex I. However, the β1-2 loop of NdhH intrudes into the PQ chamber, resulting in a smaller pocket that is occluded from the N2 cluster in our NDH-PQ structure (Fig. 5d). The occluded feature of the PQ chamber, together with the long distance between PQ and the N2 cluster, implies that our structure represents an idle state of the NDH complex, as was previously suggested for the Yl complex I^39^. This complex also possesses a β1-2 loop that intrudes into the Q chamber (Fig. 5f). The idle state of the NDH complex is presumably pivotal for its functionality in the CEF process. During this process, ferredoxin shuttles only one electron to NDH complex (PQ) at a time, which probably results in the production of an unstable semiquinone radical intermediate (PQ•H). The occluded PQ chamber may help to arrest the PQ molecule away from the N2 cluster, at least until the second electron of another Fd molecule is ready to be delivered. As a result, the PQ molecule can accept two electrons in a very short time range to produce PQH_2_. The close interaction of PQ with β1-2 loop of NdhH (Fig. 5c) may coordinate the conformational change of the β1-2 loop and a movement of PQ toward the N2 cluster. In addition, the β1-2 loop of NdhH also interacts with the TMH5–6 loop of NdhA (Fig. 5c). The TMH5 of NdhA functions in proton translocation^38^, and a number of conserved acidic residues (E226, E228, E236, E238, E239, and E240) are located in the C-terminal half of TMH5 and the TMH5–6 loop of NdhA. Therefore, changes induced by PQ movement could be further transferred to the proton pump modules, thus connecting PQ reduction and proton translocation.

## Methods

### Purification and characterization of NDH-1L

The cultivation of *T. elongatus* cells and purification of NDH-1L complex were performed according to the previous report^34^ with some modifications. The wild type (*T. elongatus* BP-1) cells from visiting Prof. Ogawa were cultured in BG-11 media at 50 °C, bubbling with CO_2_-enriched air (2%) and illuminated with white light (50–80 μmol photons m^−2^ s^−1^). Cells were harvested at logarithmic phase (OD_730_ = 0.6–0.8) by centrifugation at 5000 *g* for 5 min at 4 °C. The cell pellets were resuspended in buffer A (20 mM Bis-Tris, pH 7.0, 10 mM MgCl_2_, 10 mM CaCl_2_, 20% glycerol), and then disrupted by French press (1300 bar, four times). The lysate was centrifuged at 5000 *g* for 10 min to remove unbroken cells. The supernatant was centrifuged at 150,000 *g* for 40 min (HITACHI, rotor P45AT) to harvest thylakoid membranes. The NDH-1L complex is able to be purified through Ni^2+^-affinity chromatography owing to the native histidine-rich area within the hydrophobic subunit NdhF1. Thus the thylakoid membranes were suspended in buffer B (20 mM Bis-Tris, pH 7.0, 10 mM MgCl_2,_ 20% glycerol) and were solubilized with 1% (w/v) n-dodecyl-β-D-maltoside (β-DDM) at chlorophyll concentration of 1 mg mL^−1^. After gently stirring on ice for 1 h, the solubilized membranes were centrifuged at 35,000 *g* for 30 min to remove the insoluble material. The supernatant was loaded onto a Ni-sepharose fast flow resin (GE Healthcare) pre-equilibrated with buffer C (20 mM Bis-Tris, pH 7.0, 100 mM NaCl, 0.03% β-DDM). The resin was then washed with buffer C containing 20 mM imidazole to remove the unbound proteins. The NDH-1L complex was subsequently eluted with buffer C containing 100 mM imidazole. The eluted fraction was concentrated using a 100 kD-cut off spin concentrator. The concentrated protein was loaded onto a Superose 6 10/300 column (GE Healthcare) pre-equilibrated with buffer C. The eluted fraction containing NDH-1L was analyzed by 10–15% gradient Tris-Tricine sodium dodecyl sulfate-polyacryamide gel electrophoresis in a vertical protein gel electrophoresis system (Bio-Rad) (Supplementary Fig. 1) .

### Expression and purification of two NDH-1L subunits and Fd

The NdhS and Fd gene of *T. elongatus* were amplified by PCR from *T. elongatus* cDNA using the forward primer 5′-CGGAATTCATGATCAGGCCAATAGCTGA-3′ and reverse primer 5′-CCCAAGCTTTTACTTTTTCGCTTTCGCT-3′ for NdhS, forward primer 5′-CGGAATTCATGGCAACCTACAAAGTAA-3′ and reverse primer 5′-CCCAAGCTTTTAGTAAAGCTCTTCCTCTTGGT-3′ for Fd. The NdhV gene was artificially synthesized (GenScript, China). The three genes were constructed to vector PET-28a with N- (NdhS and Fd) or C-(NdhV) His-tags, and were all transformed into *E. coli* strain BL21 (DE3) (Transgen, China). Cells were cultured in LB media with 50 μg mL^−1^ kanamycin and shaked at 37 °C. The protein expression was induced by adding 1 mM IPTG (isopropyl β-D-1- thiogalactopyranoside) when OD_600_ reached 1.5, while Fd required extra 1 mM FeSO_4_. The cells were further incubated at 16 °C for 14 h. These three proteins were purified with the same protocol as following. The cells were harvested by centrifugation at 8000 *g* for 6 min. The cell pellets were resuspended in buffer D (25 mM Tris-HCl, pH 8.0, 300 mM NaCl, 20% glycerol, 1 mM Tris(2-carboxyethyl)phosphine (TCEP)) and disrupted by ultrasonication. The lysate was centrifuged at 35,000 *g* for 30 min, and the supernatant was loaded on to the Ni-sepharose fast flow resin (GE Healthcare), which was pre-equilibrated with buffer D. After binding for about 1 h, the resin was washed with 50 and 100 mM imidazole in buffer D, and the NdhS, NdhV, and Fd proteins were eluted with 250 mM imidazole in buffer D. The three proteins were further purified by size exclusion chromatography using a superdex 200 10/300 column (GE Healthcare) equilibrated with buffer E (25 mM Tris-HCl pH 8.0, 300 mM NaCl, 20% glycerol, 5 mM Dithiothreitol (DTT)). The fractions containing NdhS, NdhV, and Fd were collected separately and concentrated to 5 mg mL^−1^ with using 3 kDa cut off spin concentrators.

### Preparation of NDH-Fd supercomplex

The purified NDH-1L complex was concentrated to 1 mg mL^−1^ and mixed with NdhS, NdhV, and Fd proteins. The molar ratio of NDH-1L:NdhS:NdhV:Fd was set to 1:4:6:6. The mixture was incubated for 1 h at 20 °C and then concentrated to 12 mg mL^−1^ using a 100 kDa cut off spin concentrator to remove the unbound small protein. The resulted NDH-Fd sample (NDH-1L in complex with Fd) was used for cryo-EM specimen preparation.

### Grid preparation and data acquisition

Three microliter of NDH-1L (for solving the NDH-PQ structure) or NDH-Fd (for solving the NDH-Fd structure) samples at concentration of 12 mg mL^−1^ were applied to a glow-discharged holey carbon copper or gold grids (GIG, R1.2/1.3, 300 mesh). The grids were blotted for 2 s with blot force of level 2 at 95% humidity and 4 °C, and were plunge frozen into liquid ethane cooled by liquid nitrogen immediately using a semiautomatic plunge device (Thermo Fisher Scientific Vitrobot IV). Sample screen was performed using Talos F200C 200 kV or Talos L120C 120 kV electron microscopes equipped with a Ceta camera (Thermo Fisher Scientific). For structure determination, the micrographs of both NDH-PQ and NDH-Fd were collected using SerialEM software suite^55^ on a 300 kV Titan Krios electron microscope (Thermo Fisher Scientific) equipped with a K2 camera (Gatan) and a GIF quantum energy filter (20 eV). Total of 1463 images for NDH-PQ and 2090 micrographs for NDH-Fd were acquired using a defocus range between 0.5 and 2.5 μm at 130,000 magnification. The micrographs were exposed for 10 s and dose fractionated into 32 frames, leading to a total dose of 60 e^−^ Å^−2^.

### Image processing

Beam-induced motion of each movie stack was corrected by software MotionCor2^56^, and the parameter of the contrast transfer function on each micrograph was determined by the program CTFFIND4^57^. The software Relion-3.0 was used for further data processing^58^. For the NDH-PQ sample, about 1000 particles were manually picked and processed with reference-free 2D classification. Five class-averaged images were selected as references for subsequent particle auto-picking. A total of 434,021 particles were picked from 1463 micrographs. After 2D classification, 346,668 particles in good classes were kept for further data processing. A reference map was generated by the 3D-initial-model program in Relion-3.0 and the particles were classified into four classes by 3D classification. Class 1 containing 139,095 particles, is a NDH-PQ monomer, while class 3 containing 48,693 particles, is a NDH-PQ dimer. After refinement, the structures of NDH-PQ in either monomer or dimer showed the same conformational and structural features. The particles from the two classes were merged together for 3D autorefinement with a soft mask to mask monomer and resulted in a 3.3 Å density map. After CTF refinement and another round of 3D autorefinement, the final resolution of NDH-PQ density map was improved to 3.0 Å estimated based on the gold-standard Fourier shell correlation with 0.143 criterion (Supplementary Fig. 2). The data of NDH-Fd sample were processed in the same way as mentioned above. A total of 276,859 particles were picked from 2090 micrographs. After 2D classification, 246,088 NDH-Fd monomer particles were kept for 3D classification with the NDH-PQ map as the initial model. 152,003 particles were selected for further 3D autorefinement and resulted in a 3.20 Å density map after CTF and beam-tilt refinement and beam-tilt estimation. To further improve the density of Fd, NdhV, and NdhS, focused refinement was used with a soft edged mask around the peripheral region, which resulted in resolution of 3.15 Å of the peripheral region (Supplementary Fig. 3). The local resolution of the final map was calculated using ResMap^59^.

### Model building and refinement

For the model building of subunits conserved in respiratory complex I (NdhA–NdhK), the homologous models were generated on the basis of the crystal structure of *T. thermophilus* complex I (PDB ID: 4HEA)^38^ by using software Chainsaw in the CCP4 software suite^60^. These homologous models were manually fitted in to the 3.0 Å cryo-EM map of NDH-PQ one by one using UCSF chimera^61^. The OPS subunits NdhL–NdhQ were built and adjusted manually by Coot^62^ on the basis of the protein sequence and the predicted secondary structures. In the cryo-EM map of NDH-PQ, the density of NdhS subunit is very weak and the density of NdhV is invisible, but they behave well in the cryo-EM map of NDH-Fd complex. Therefore the de novo model building of NdhS and NdhV were performed on the basis of the 3.15 Å cryo-EM map of NDH-Fd complex. Regarding Fd protein, the crystal structure of Fd from *T. elongatus* BP-1 (PDB ID: 5AUI)^63^ was manually fitted in to 3.15 Å cryo-EM map of NDH-Fd using UCSF chimera. The initial models of NDH-PQ and NDH-Fd were refined in PHENIX-v1.15^64^ using phenix.real_space_refine with geometry and secondary structure restraints. During real-space refinement, the distances between the iron atoms in the iron/sulfur clusters and the sulfur atom in the cysteine surrounded iron/sulfur clusters were constrained according to the values obtained from the high-resolution crystal structures. Automatic real-space refinements followed by manual correction in Coot were carried out interactively. The geometries of the final structures were assessed using MolProbity^65^. High-resolution images for publication were prepared using UCSF chimera and PyMOL (Molecular Graphics System, LLC).

### Measurement of NDH activity in vivo

The postillumination increase in chlorophyll fluorescence was used to ascribe NDH-1L activity. The chlorophyll fluorescence was detected by a pulse-amplitude-modulation (PAM) chlorophyll fluorometer (Walz, Effeltrich, Germany), with emitter-detector-cuvette assembly (ED-101US), and an 101 ED unit as described by Schreiber et al.^66^. The parameter Fo is a minimal fluorescence, which reflects the yield of Chl fluorescence when the reaction center is fully opened, and can be used to reflect the redox state of the primary electron acceptor PQ (QA). The transient increase of Fo after termination of the actinic light is used to represent a reduction of PQ by photoreductants accumulated in cytosol during illumination, which is used to indirectly reflects the CEF activity of NDH. Cells were harvested at logarithmic phase (OD_730_ = 0.6–0.8), and suspended in different pH buffer, pH 6.0 (20 mM HEPES), pH 7.0 (20 mM HEPES), pH 8.0 (20 mM Tris), pH 9.0 (20 mM Glycine), pH 10.0 (20 mM Glycine) before detected with PAM, and incubated in dark for 5 min before measurement. Each experiment was independently repeated for four times and standard deviations were calculated.

### SPR-based biosensor analysis

The interaction of the NDH-1L with Fd was detected by SPR using a Biacore T100 biosensor (GE Healthcare). The experiment was performed as described by He et al.^34^ with some modifications. NDH-1L was diluted with 10 mM Glycine, pH 4.5 to the concentration of 50 μg mL^−1^, and then immobilized on the activated CM5 chip surface to about 5000 RU. The noncovalently protein was removed with 2 M NaCl. To compare the different interactions between NDH-1L and Fd in various pH conditions, three running buffers (50 mM NaH_2_PO_4_, 0.03% β-DDM) with different pH (pH 6.0, pH 7.0, and pH 8.0) were prepared. A series of concentration of Fd (0.625, 1.25, 2.5, 5, 10, and 20 μM) diluted separately with three running buffers were injected using a flow rate of 30 μL min^−1^ for 1 min. After 1 min dissociation, the bound Fd was washed off withv 2 M NaCl. The data were analyzed with Biacore T100 evaluation software (GE Healthcare) by fitting to a 1:1 binding model.

### Reporting summary

Further information on research design is available in the Nature Research Reporting Summary linked to this article.

## Supplementary information

Supplementary Information

Reporting Summary

## Data Availability

Data supporting the findings of this paper are available from the corresponding authors upon reasonable request. A reporting summary for this article is available as a Supplementary Information file. The source data underlying Supplementary Figs 1e, f, 7, and 9c are provided as a Source Data file. The cryo-EM density maps have been deposited in the Electron Microscopy Data Bank (EMDB) under accession codes EMDB-9989 (for NDH-Fd structure) and EMDB-9990 (for NDH-PQ structure), and model coordinates have been deposited in the Protein Data Bank under accession number 6KHI (for NDH-Fd structure) and 6KHJ (for NDH-PQ structure).

## Source data

Source Data
