# Supplementary material for: Structural basis for electron transport mechanism of complex I-like photosynthetic NAD(P)H dehydrogenase
Source: Nat Commun. 2020 Jan 30;11:610. doi: 10.1038/s41467-020-14456-0 (PMC6992706; doi:10.1038/s41467-020-14456-0)
Supplement: Supplementary file 1 — Supplementary Information [file 41467_2020_14456_MOESM1_ESM.pdf]

Structural basis for electron transport mechanism of complex I-like photosynthetic NAD(P)H dehydrogenase

Xiaowei Pan[1], Duanfang Cao[1], Fen Xie[1,2], Fang Xu[2,3], Xiaodong Su[1], Hualing Mi[3], Xinzheng Zhang[1,2,4], Mei Li[1]

Affiliations:

[1]National Laboratory of Biomacromolecules, CAS Center for Excellence in Biomacromolecules, Institute of Biophysics, Chinese Academy of Sciences, Beijing 100101, P.R. China.

[2]University of Chinese Academy of Sciences, Beijing 100049, P.R. China.

[3]National Key Laboratory of Plant Molecular Genetics, Institute of Plant Physiology and Ecology, Shanghai Institutes for Biological Sciences, Chinese Academy of Science, Shanghai, 200032, P.R. China.

[4]Center for Biological Imaging, CAS Center for Excellence in Biomacromolecules, Institute of Biophysics, Chinese Academy of Sciences, Beijing 100101, P.R. China.

These authors contributed equally: Xiaowei Pan, Duanfang Cao, Fen Xie, Fang Xu

Correspondence and requests for materials should be addressed to H.M. (email:hlmi@sibs.ac.cn) or to X.Z. (email: xzzhang@ibp.ac.cn)or to M.L. (email: meili@ibp.ac.cn)

Supplementary Figures

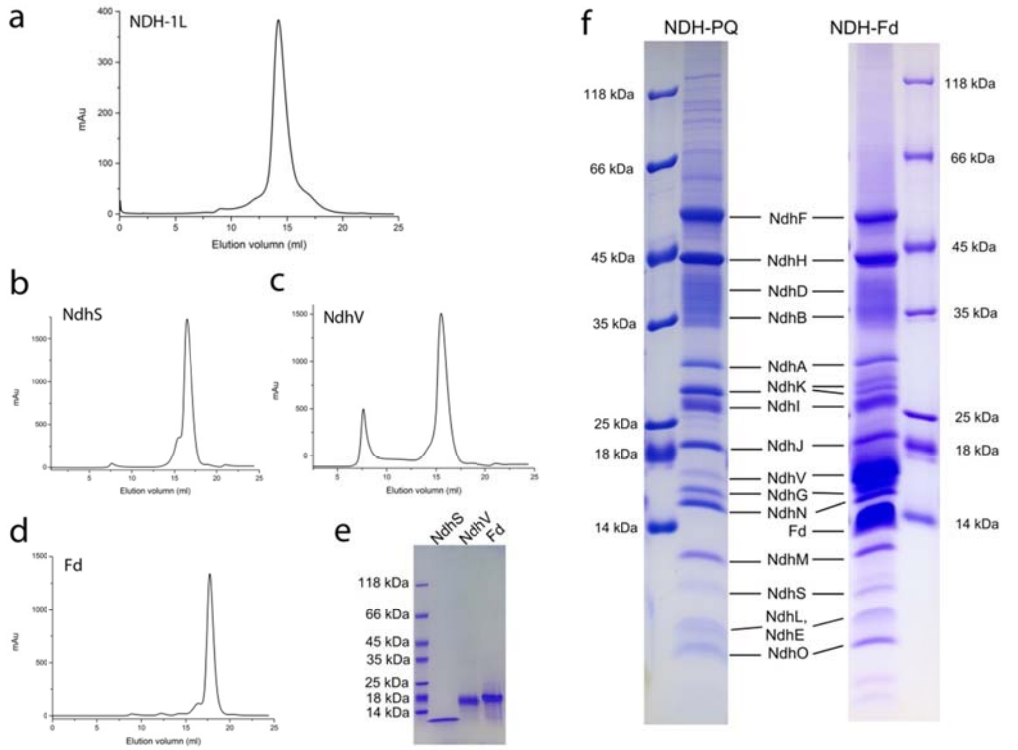

Supplementary Figure 1 Preparation and characterization of NDH-1L supercomplex as well as Fd, NdhV and NdhS from *T. elongatus*.

(a-d) Size-exclusion chromatographic curves of NDH-1L supercomplex (a), NdhS (b), NdhV (c) and Fd (d). (e) SDS-PAGE analysis of purified NdhS, NdhV and Fd proteins. (f) SDS-PAGE analysis of NDH-1L supercomplex (used for solving the NDH-PQ structure) and NDH-1L supercomplex incubated with purified NdhS, NdhV and Fd proteins (used for solving the NDH-Fd structure). The protein composition of each Coomassie band was indicated based on the mass spectrometry and proteomics data analysis. Source data of (c) and (d) are provided as a Source Data file**.**

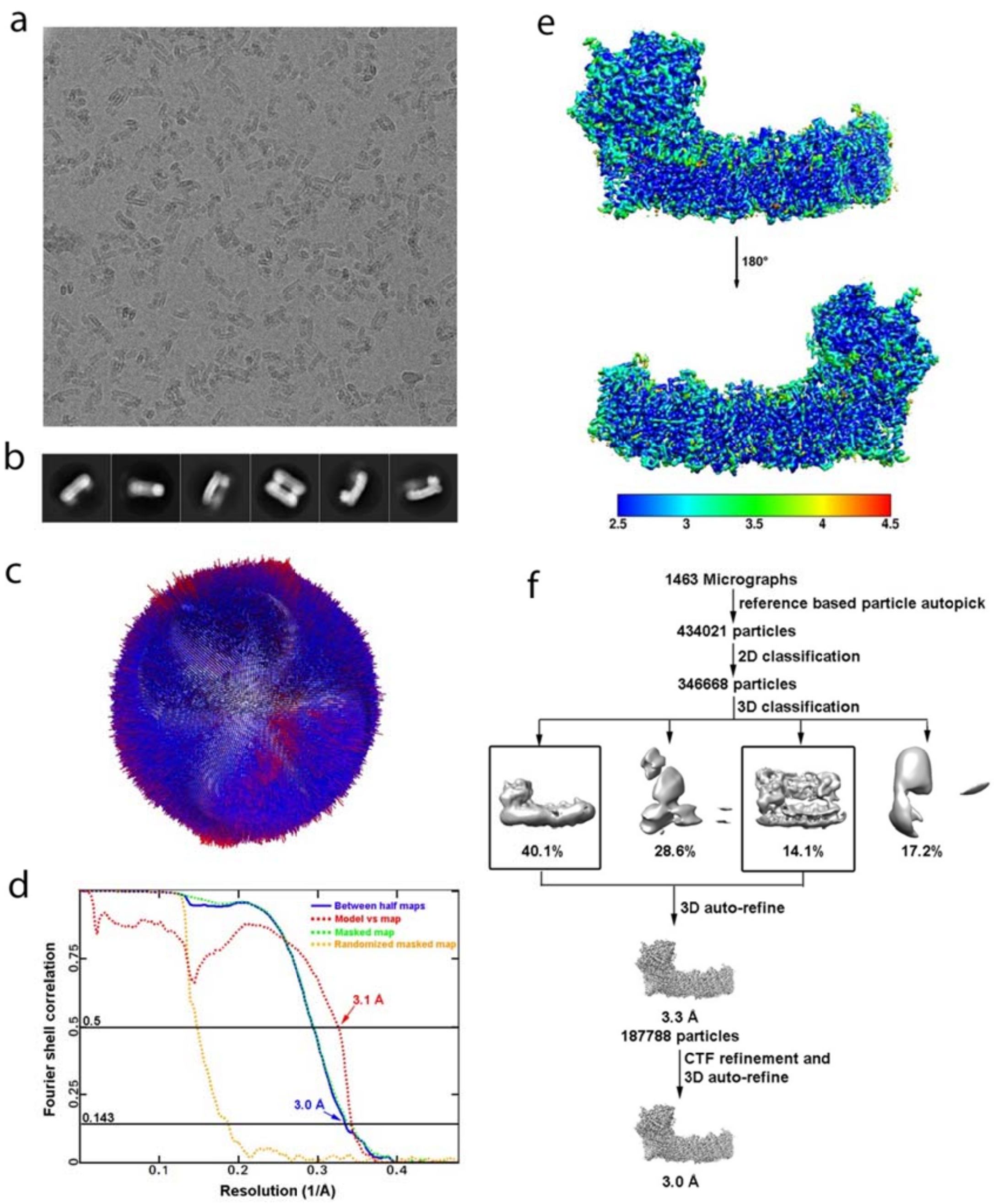

Supplementary Figure 2 Cryo-EM analysis of NDH-PQ complex.

(a) A representative cryo-EM micrograph of NDH-PQ complex. (b) Representative 2D class averages in the final round of reference free 2D classification. (c) Angular distributions of the particles in the final 3D auto-refinement. (d) Fourier shell correlation (FSC) of the overall NDH-PQ complex calculated between the two cryo-EM half-maps (blue solid line, with a value of 0.143 at 3.0 Å) and the FSC calculated between the refined structure model and the final cryo-EM map (red dotted line, with a value of 0.5 at 3.1 Å), FSC of the masked map (green dotted line) and FSC of the randomized masked map (orange dotted line). (e) Local resolution maps of the NDH-

PQ complex estimated by Resmap. (f) Image processing workflow of NDH-PQ complex.

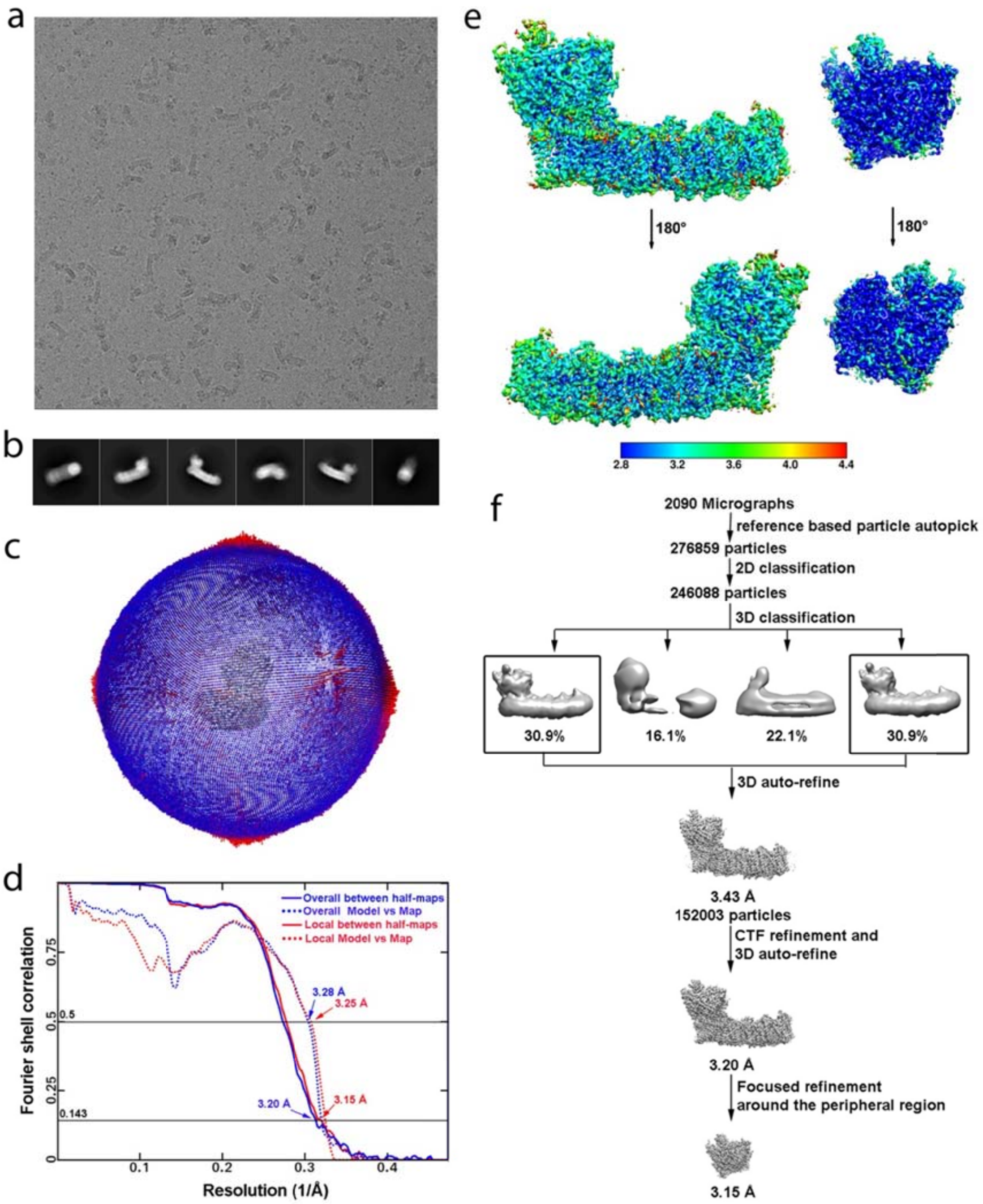

Supplementary Figure 3. Cryo-EM analysis of NDH-Fd complex.

(a) A representative cryo-EM micrograph of NDH-Fd complex. (b) Representative 2D class averages in the final round of reference free 2D classification. (c) Angular distributions of the particles in the final 3D auto-refinement. (d) Fourier shell correlation (FSC) of the NDH-Fd complex calculated between the two cryo-EM half-maps (blue solid line, the FSC of overall NDH-Fd complex with a value of 0.143 at 3.20 Å; Red solid line, the FSC of local refined NDH-Fd peripheral region with a value of 0.143 at 3.15 Å) and the FSC calculated between the refined structure model

and the final cryo-EM map (blue dotted line, the FSC of NDH-Fd complex with a value of 0.5 at 3.28 Å; red dotted line, the FSC of local refined NDH-Fd peripheral region with a value of 0.5 at 3.25 Å). (e) Local resolution maps of the overall NDH-Fd complex (left) and the local refined NDH-Fd peripheral region (right) estimated by Resmap. (f) Image processing workflow of NDH-Fd complex.

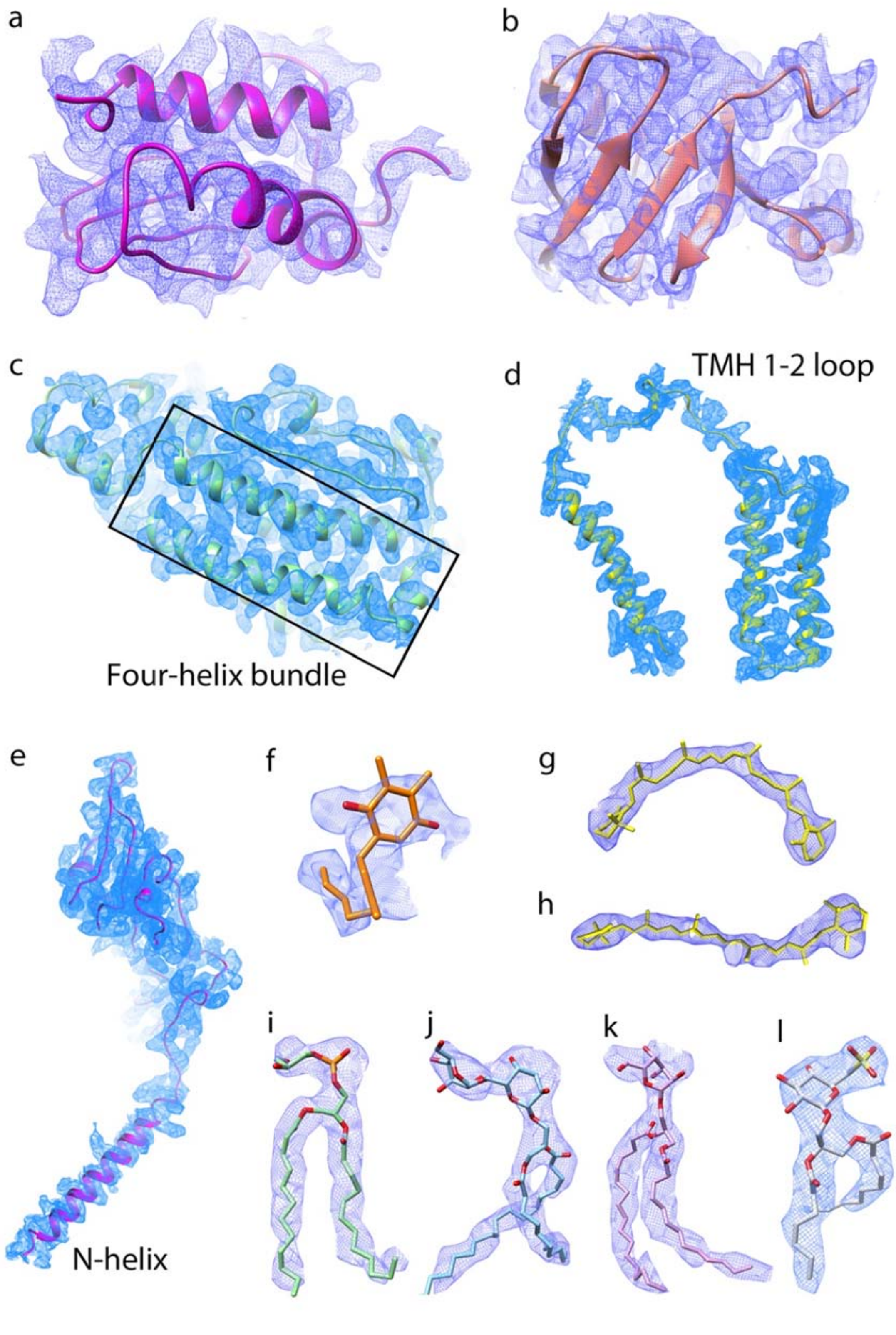

Supplementary Figure 4 Representative cryo-EM densities.

(a-e) Cryo-EM densities of protein subunits NdhV (a), Fd (b), NdhH (c), NdhC (d) and NdhI (e). (f-k) Cryo-EM densities of cofactors PQ (f), BCR1 (g), BCR2 (h), and

lipids phosphatidyl glycerol (PG) (i), digalactosyldiacyl glycerol (DGDG) (j), monogalactosyldiacyl glycerol (MGDG) (k) and sulfoquinovosyldiacyl glycerol (SQDG) (l).

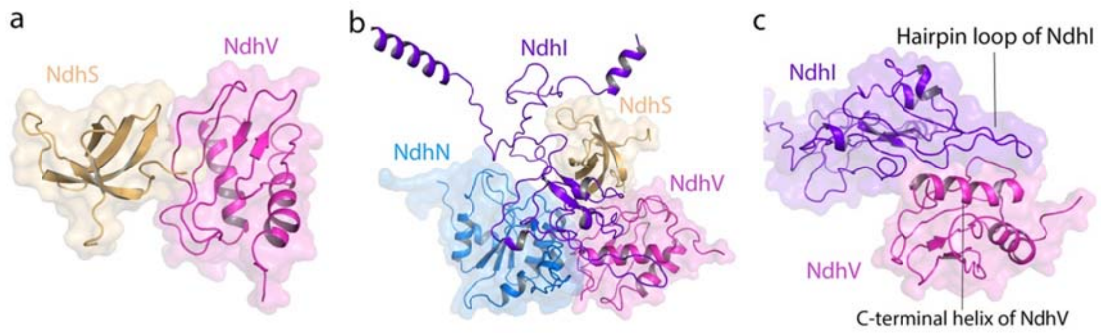

Supplementary Figure 5 Interaction between NdhV and other subunits.

(a) NdhV and NdhS form strong interactions. (b) NdhV, NdhS and NdhN form a triangular structure, contacting each other and interacting with NdhI. (c) NdhV interacts with the hairpin loop of NdhI.

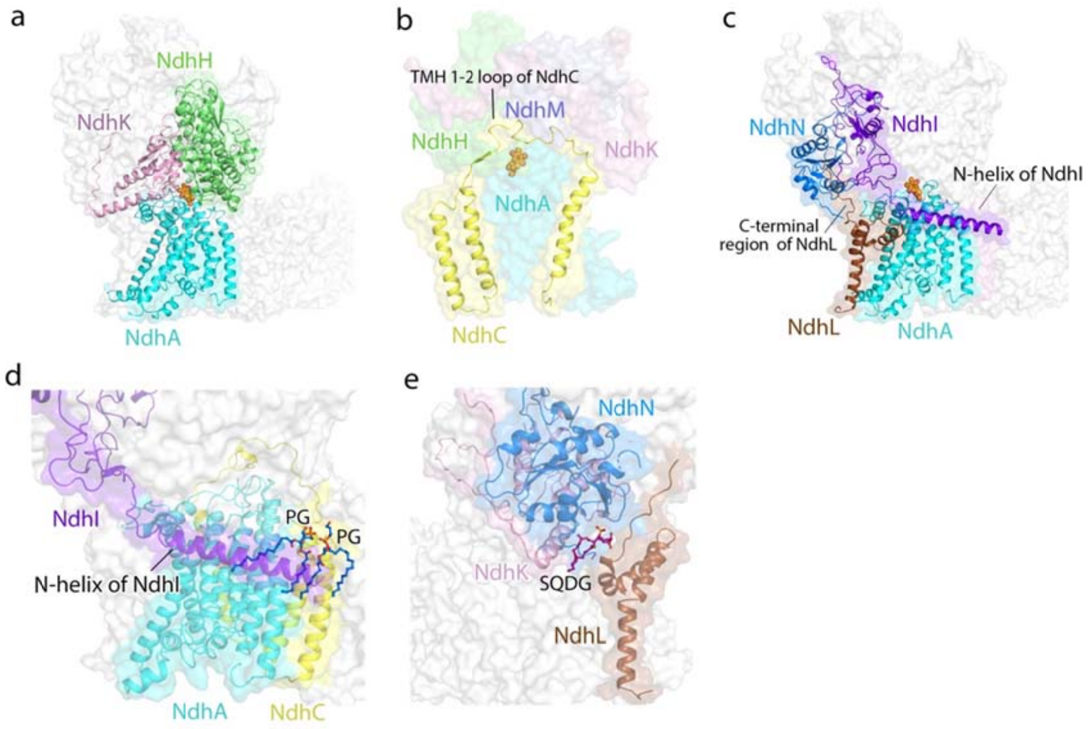

Supplementary Figure 6 Interactions between the membrane arm and the hydrophilic arm of NDH-1L complex.

(a) The membrane subunit NdhA and the hydrophilic subunits NdhH and NdhK are the major structural elements essential for the association between the membrane and hydrophilic arms. (b) The TMH1-2 loop of NdhC simultaneously interacts with the membrane subunit NdhA and the hydrophilic subunits NdhH, NdhK and NdhM. (c) The C-terminal loop of NdhL interacts with hydrophilic subunits, whereas the N-helix of NdhI contacts the membrane arm. Subunits involved in the interactions are showing in cartoon mode and colored differently. The PQ molecule are shown as spheres in (a-c). (d) Two lipids are sandwiched between the N-helix of NdhI and the membrane domain of NdhA and NdhC. (e) One lipid molecule simultaneously interacts with NdhL, NdhN and NdhK.

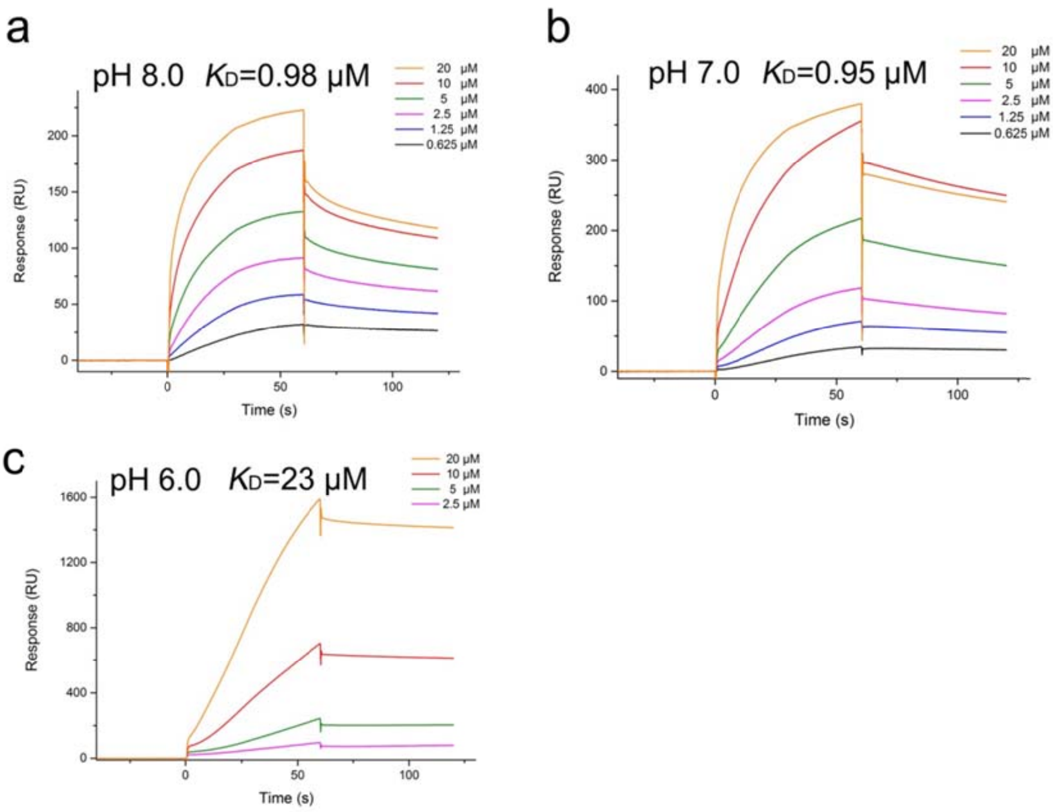

Supplementary Figure 7 The binding affinity between Fd and NDH-1L under different pH conditions.

(a-c) SPR analysis of the binding of Fd with NDH-1L complex at pH 8.0 (a), 7.0 (b) and 6.0 (c). The Fd concentrations and the binding constant (K_D) in different pH conditions are indicated. Source data are provided as a Source Data file.

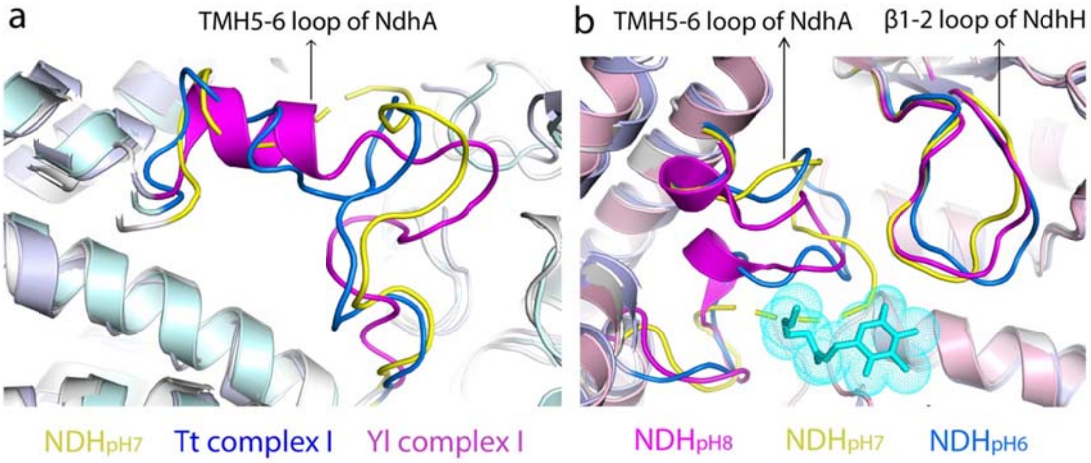

Supplementary Figure 8 Structural comparison of the flexible loops in NDH-1L. and complex I. (a) Comparison of the TMH5-6 loops of NdhA in NDH_{pH7} (NDH-PQ) structure with the corresponding structural elements of complex I structures (PDB codes: 4HEA for Tt complex I, 4WZ7 for Yl complex I). (b) Comparison of the TMH5-6 loops of NdhA and β1-2 loops of NdhH among three structures of NDH-1L complex purified at different pHs.

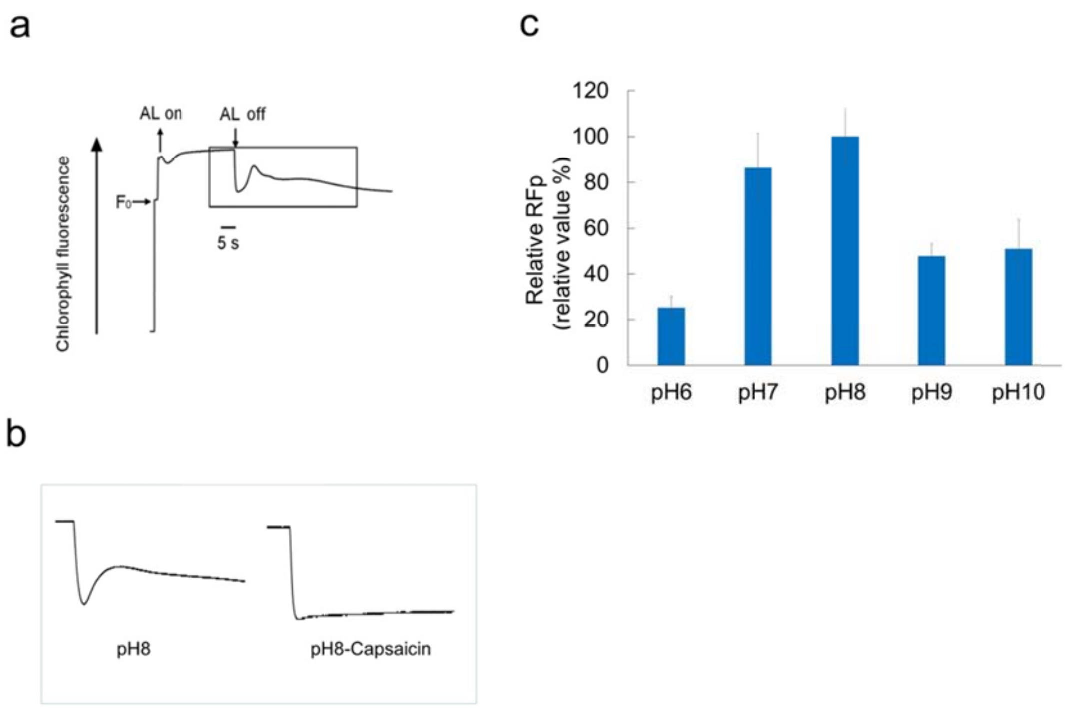

Supplementary Figure 9 CEF activity of NDH-1L under different pH conditions. Monitoring of the activities of cyclic electron flow around photosystem I (PS I) mediated by NDH-1 of *Thermosynechococcus elongatus* cells using chlorophyll (Chl) fluorescence analysis. (a) A typical Chl fluorescence kinetics of the cyanobacterial cells. Fo, minimal fluorescence, which reflects the yield of Chl fluorescence when the reaction center is fully opened, and can be used to reflect the redox state of Q_A. The transient increase of Fo after termination of the actinic light is used to represent a reduction of PQ by photoreductants accumulated in cytosol during illumination, and is used to indirectly reflects the CEF activity of NDH-1L. The boxed area shows kinetics of the transient increase in Chl fluorescence after the actinic light (AL) turning off. (b) The effect of 400 μmol capsaicin, a specific inhibitor of NDH-1 on the kinetics of the transient increase in Chl fluorescence. The cells with capsaicin do not show Chl fluorescence increase (right) compared with the capsaicin-free cells (left). (c) CEF activity of NDH-1L at different pHs shown by the relative rate of post-illumination increase in Chl fluorescence (RFp). The mean value and standard errors were calculated from four independent measurements under each pH conditions.
